# Guyon's canal syndrome due to tortuous ulnar artery with DeQuervain stenosing tenosynovitis, ligamentous injuries and dorsal intercalated segmental instability syndrome, a rare presentation: a case report

**DOI:** 10.1186/1757-1626-2-9390

**Published:** 2009-12-23

**Authors:** Muhammad Zeeshan, Farhan Ahmed, Darakhshan Kanwal, Qazi Saad Bin Khalid, Muhammad Nadeem Ahmed

**Affiliations:** 1 Department of Diagnostic Radiology, Aga Khan University Hospital, Stadium Road, P O Box 3500, Karachi, Pakistan

## Abstract

The Guyon's canal syndrome is a well known clinical entity and may have significant impact on patient's quality of life. We report a case of 43-year-old male who presented with complaints of pain and numbness in right hand and difficulty in writing for past one month. On imaging diagnosis of Guyon's canal syndrome because of tortuous ulnar artery was made with additional findings of DeQuervain's stenosing tenosynovitis and dorsal intercalated segmental instability syndrome with ligamentous injury and subsequently these were confirmed on surgery.

Although it is a rare syndrome, early diagnosis and treatment prevents permanent neurological deficits and improve patient's quality of life.

## Introduction

The Guyon's canal is a fibro-osseous tunnel situated along the anteromedial aspect of wrist joint. It contains the ulnar nerve, ulnar artery and veins [1]. Its main clinical significance is because of potential ulnar nerve compression, can occur while nerve traversing this canal [1].

It can be caused by a number of causes like trauma [2], lipoma [3], ganglion cysts [4], and anatomic variants such as presence of abductor digiti minimi muscles [5], coursing via the canal, or presence of a fibrous arch overlying the deep motor branch of the ulnar nerve.

Rare causes due to arterial compression include posttraumatic pseudoaneurysm, true aneurysm of distal ulnar artery or tortuous ulnar artery [6,7].

Only two case reports of tortuous ulnar artery leading to compression of ulnar nerve in Guyon's canal yet have been published in the literature while no one associated with other incidental findings as seen in our case.

## Case presentation

A 43-year-old male Asian, Pakistani presented in our outpatient department with complaints of pain and numbness in right hand and difficulty in writing, for past one month. On further questioning, there was a remote history of fall while playing hockey. On examination, the bulk and tone of right hand was normal, however, the power was decreased to 3/5.

**Figure 1:**
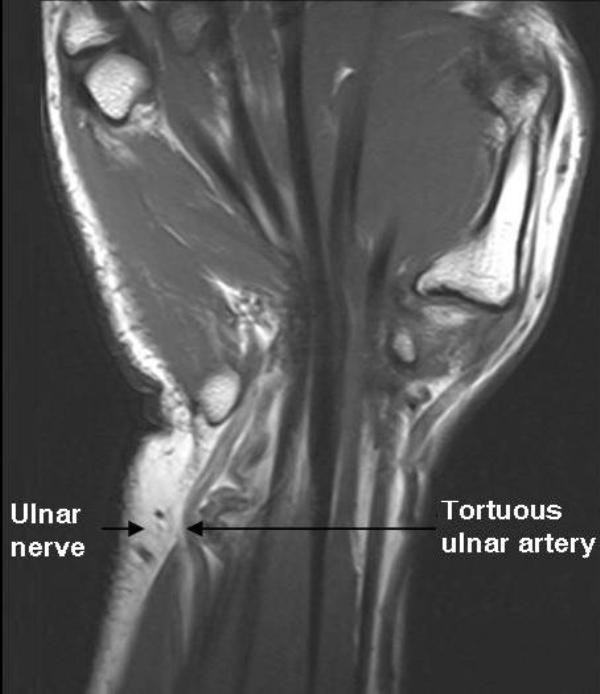
**Coronal MRI image: Ulnar nerve compression in Guyon's canal due to tortuous ulnar artery**.

Subsequently, an x-ray of right hand was ordered which was normal. As the signs and symptoms of the patient were not improving on previous medications, MRI of right hand was advised, which revealed tortuous distal ulnar artery just proximal to the wrist joint causing compression of ulnar nerve within Guyon's canal (Figure 1). Abnormal signal intensity was also seen in nerve with its mild post gadolinium enhancement. On the basis of these findings, diagnosis of Guyon's canal syndrome was made.

**Figure 2:**
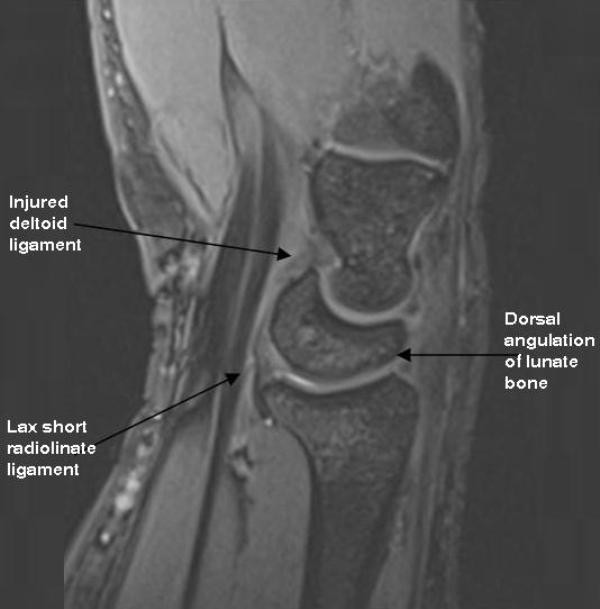
**Sagittal MRI image: Ligamentous injuries leading to dorsal angulation of lunate bone - dorsal intercalated segment instability (DISI)**.

In addition, there was inflammation of first extensor compartment with thickening of abductor pollicis longus and extensor pollicis brevis, suggestive of De Quervain's stenosing tenosynovitis. The tendon sheath of extensor carpi ulnaris was also inflamed. There was dorsal tilt of lunate bone with angle measuring greater than 30 degrees with associated mild dorsal placement of capitate bone in relation to the radius. Mild increased signal intensity seen within the short radiolunate ligament and deltoid/arcuate ligament with also evidence of laxity seen. Considering these findings, the diagnosis of dorsal intercalated segmental instability syndrome (DISI) with ligamentous injuries were also made (Figure 2).

The patient then underwent right ulnar nerve decompression, excision of torn ligaments with arthrodesis and closing wedge osteotomy of 1^st ^metacarpal bone.

He was then followed as an outpatient and is doing well, except for mild loss of sensation over the dorsal aspect of skin of right thumb. He regained his power 5/5 in his right hand, with the exception of right little finger, power of which remains 4/5.

## Discussion

The medial cord of brachial plexus gives origin to ulnar nerve (C8 and T1). The ulnar nerve descends along the axillary and brachial arteries medially and downward up to the mid shaft of humerus. The nerve then courses dorsally, penetrates the medial intermuscular septum and descends along medial head of triceps muscle. It then enters into the cubital tunnel, at the medial condyle of humerus [8].

Below the elbow, it descends between two heads of flexor carpi ulnaris muscle and courses distally between the flexor carpi ulnaris and the flexor digitorum profundus muscles. At wrist, the ulnar nerve runs through the Guyon's canal.

The neuropathies produced by entrapment of the ulnar nerve include cubital tunnel syndrome and Guyon's canal syndrome. It results from a lesion of the ulnar nerve at the level of the Guyon canal (also called the pisohamate tunnel) [9].

The possible causes of ulnar nerve lesions at the Guyon canal include ganglia, lipomas, and other cysts; anomalies of ligaments or muscles; ulnar artery aneurysms; fractures of the radius, pisiform bone, hook of the hamate, or other wrist bones; and chronic repetitive trauma [2-7].

The MRI is the best imaging modality to diagnose and classify these lesions, especially the T1 weighted sequences. On T1-weighted images, the nerve can be seen as a round or ovoid structure with surrounding small amount of fat. The ulnar nerve bifurcation is usual well visualized and can be followed distally [10].

The size and signal intensity of ulnar nerve is an important marker and should be assessed in patients suspected to have ulnar nerve lesions in the Guyon's canal. MR imaging may help to exclude mass lesion and can also demonstrate compression by an anomalous or accessory muscle or fibrous band [10]. In addition, associated abnormalities of intrinsic hand muscles and ligaments can also be detected.

The DeQuervain's tenosynovitis is more common in females and presents with pain and swelling in the first dorsal extensor sheath. The female gender, greater than 40 years of age and black race are the predisposing factors [11]. It is usually diagnosed clinically, however, different authors have recommended different imaging modalities, like magnetic resonance imaging, ultrasound, and bone scanning [12].

On MRI, the most reliable sign to diagnose DeQuervain's synovitis is increased thickness of extensor pollicis brevis and abductor pollicis longus tendons with peritendinous edema. Surrounding subcutaneous edema and increased intratendinous signals may support the diagnosis [13].

Recent advances in MR imaging showed that DISI and VISI can be diagnosed on sagittal images, as on plain x-ray film malposition of wrist can mimic these in certain cases. The capitolunate, radiolunate and scapholunate angles are used to diagnose it [14].

In literature, there are only two cases reported yet and none in any of the radiology journal. In the case, reported by Segal et al [15]. patient presented with paresthesia, tingling and burning sensations of hand without any motor symptoms while in the case reported by Emel et al [1], there were both sensory and motor symptoms with permanent deficits even after decompression.

In our case, both sensory and motor symptoms were the presenting complaint and after decompression patient still has some sensory and motor deficits.

## Conclusion

The tortuous peripheral arteries are very rare causes of peripheral nerve entrapment syndromes. The MRI is an excellent tool of diagnosis in such cases and also delineates other important associated findings. The early diagnosis and subsequent treatment prevent permanent deficits and improve patient's quality of life.

## Competing interests

The authors declare that they have no competing interests.

## Authors' contributions

MZ: participated in literature search, manuscript writing and review of document.

FA: was the radiologist involved in diagnosis, contributed in literature search and manuscript review.

DK: participated in literature search, reviewing the document and coordination with the patient.

QSBK: participated in manuscript review, collecting imaging and surgical data.

MNA: was senior radiologist involved in the diagnosis.

All authors read the paper and approved the final manuscript.

## Consent

Written informed consent was obtained from the patient for publication of this case report and accompanying images. A copy of the written consent is available for review by the Editor-in-Chief of this journal.
